# Usefulness of videoconferencing in psychiatric emergencies -- a qualitative study

**DOI:** 10.1007/s12553-017-0189-z

**Published:** 2017-03-24

**Authors:** Stein Roald Bolle, Marianne Vibeke Trondsen, Geir Øyvind Stensland, Aksel Tjora

**Affiliations:** 1 Norwegian Centre for eHealth Research, University Hospital of North Norway, P.O. Box 35, N-9038 Tromsø, Norway; 2 Division of Emergency Medical Services, University Hospital of North Norway, P.O. Box 45, N-9038 Tromsø, Norway; 3 Division of Surgical Medicine and Intensive Care, University Hospital of North Norway, P.O. Box 45, N-9038 Tromsø, Norway; 4 Division of Mental Health and Substance Abuse, General Psychiatric Clinic, University Hospital of North Norway (UNN), P.O. Box 6124, N-9291 Tromsø, Norway; 5 Department of Sociology and Political Science, Norwegian University of Science and Technology, N-7491 Trondheim, Norway

**Keywords:** Psychiatry, Emergency care, Videoconferencing, Tele-psychiatry, Qualitative study

## Abstract

High quality health services for psychiatric emergencies are difficult to maintain 24 h a day in sparsely populated areas, where the availability of specialists are limited. To overcome this challenge, the University Hospital of North Norway implemented an on-call system in psychiatric emergencies, by which psychiatrists are accessible 24/7 for telephone and videoconferencing consultations with patients and nurses at three regional psychiatric centers. The purpose of this study was to explore the usefulness of videoconferencing consultations in psychiatric emergencies. The on-call psychiatrists and nurses at the regional psychiatric centers who participated in at least one videoconferencing consultation were recruited for semi-structured interviews: 5 psychiatrists and 19 nurses for a total of 24 participants. The interviews were performed from July of 2012 to June of 2013. The interviews were transcribed and analyzed using a stepwise-deductive-inductive approach supported by the HyperRESEARCH analysis software. After the introduction of the videoconferencing system, telephone consultations were still used for straightforward situations when further treatment or admission decisions were obvious. Videoconferencing consultations, during which patients were present, were useful for challenging situations when there was 1) uncertainty regarding the degree of illness or the level of treatment, 2) a need to clarify the severity of the patient condition, 3) a need to build an alliance with the patient, and 4) disagreement either between health personnel or between the patient and health personnel. Videoconferencing is useful for challenging and complex psychiatric emergencies and is a suitable tool for building high quality, decentralized psychiatric services.

## Introduction

Psychiatric emergencies require 24/7 health services for all patients, including those in remote or sparsely populated areas. Decentralization of psychiatric health services [1] has led to challenges in maintaining a sufficient number of clinical experts outside larger cities [2, 3]. When psychiatric emergencies occur in locations far from psychiatric expertise, patients are often transported to specialized hospital wards for assessment, treatment and care. Commonly patients report traumatic experiences related to long travel distances and new environments when they are in such vulnerable situations. There is a need for exploring new ways of organizing and delivering emergency psychiatric services, aiming to provide high-quality services in areas lacking psychiatric expertise [4].

Videoconferencing (VC) may be a tool to replace physical co-location of patient and provider and has been used for decades for advanced psychiatric consultations in remote areas [3, 5–7]. A broad range of mental health issues has been successfully handled by the use of VC [2, 3, 5, 8]. Patients are satisfied with such services, due to increased access to therapy, quality of the consultation, saved time and reduced travel needs [2, 3, 9]. In pediatric mental health services, children and youth even have stated that use of VC technology in therapy alleviated their anxiety regarding consulting a psychiatrist [2]. From the perspective of frontline mental health workers, access to psychiatric expertise through VC were reported to enhance the capacity of mental health services in remote and rural areas, by increased knowledge, competence and feeling of confidence in assisting their patients [5]. For psychiatric emergencies, however, there is limited experience and research on the use of VC. Psychiatric emergencies often require team decisions, for which videoconferencing may be the right tool when patients and health personnel are not co-located. Although experience with emergency telepsychiatry is limited, it is found to be safe, has improved patient assessment and care in acute situations, and reduced unnecessary admissions in emergency psychiatric wards [10].

The University Hospital of North Norway installed the first videoconferencing system for psychiatric emergencies in Norway, as a mean to solve the need for psychiatric expertise in sparsely populated areas. This system allows patient-provider communication through video consultations in which patients, local nurses and remote psychiatrists participate [11]. In a previous paper, we reported that patients and health personnel found that this system led to an increased confidence in psychiatric emergencies, and that patients were more involved through VC when diagnoses and treatment plans were made [12]. However, more knowledge is needed regarding the usefulness of VC for psychiatric emergencies [10, 13, 14]. This paper explores situations in which health personnel find VC useful for patients in psychiatric emergencies.

## Material and methods

### Site of research

The Department South of the Division of Mental Health and Substance Abuse at the University Hospital of North Norway serves 95,000 people living in the surrounding towns and rural communities of Tromsø, Norway. The department has three regional psychiatric centers (RPCs) located in towns with driving distances 2.5 to 5 h from the acute psychiatric hospital ward in Tromsø. The RPCs are responsible for delivering psychiatric emergency services in their geographical areas. Each of the RPCs has a ward with 12 beds, an ambulatory psychiatric team, and outpatient services. Involuntary admissions can only be handled by the acute ward in Tromsø. Because of the geographical remoteness of the RPCs, positions for psychiatrists have been vacant and it has not been possible to provide regular 24-h emergency care. To overcome this challenge, in 2011 the Division implemented a new decentralized on-call system for psychiatric emergencies in collaboration with the Norwegian Centre for Integrated Care and Telemedicine [11]. Six psychiatrists were organized in a rotation scheme and accessible for the three RPCs by either telephone or VC. The purpose of the new on-call service was to ensure that all patients had equal access to specialist assessments independent of their location. VC studios were installed at the three RPCs and in the offices or homes of six psychiatrists. The psychiatrists agreed to be available for direct patient consultations by VC in collaboration with local nurses in order to provide a 24/7 emergency service.

### Research design and participants

A qualitative exploratory design was used comprising semi-structured audio-recorded interviews with patients and health personnel who had participated in at least one VC consultation. Interviews were conducted individually with 24 participants: 5 of the 6 on-call psychiatrists (5 males, ages 40 to 60) and 19 nurses from the three RPCs (4 males and 15 females, ages 39 to 63). The nurses were specially trained in psychiatric healthcare and were experienced in handling psychiatric crises. The nurses generally worked autonomously and only contacted the on-call psychiatrists when they needed advice or help evaluating the patient.

The interviews were conducted one to two years after the VC system became operational in 2011. The interviews focused on the participants’ experiences using VC consultations to provide emergency psychiatric care. Interview guides structured the dialogue, tailored to each of the target groups; the on-call psychiatrists and the nurses at the RPCs. The interviews with the psychiatrists lasted between 65 and 80 min. Since the interviews of nurses were aiming at detailed description of VC experiences, these interviews varied significantly in length, between 34 and 75 min, depending on their amount of experience with VC consultations. The interviews were transcribed verbatim.

### Data generation and analysis

The analysis of the transcripts followed a stepwise-deductive-inductive (SDI) approach [15] directed towards inductively identifying issues and themes across the empirical material. Such issue-focused analysis implies focusing on emerging topics, not on individuals, and interview data from some informants might contribute more to the analysis than data from others [16]. According to the SDI approach, our aim was to explore types of issues raised during the interviews, to identify experiences of using VC in psychiatric emergencies.

Empirically close coding and sorting of the codes are the first steps in the SDI approach. First, the transcripts were coded by using the HyperRESEARCH analysis software (version 3.5.2, ResearchWare Inc., 2013), maintaining the very detailed interview content within code names. This work resulted in 241 codes regarding the users’ experiences. These codes were sorted into nine “code groups” [15] on basis of coherence within each group. Since some of these groups were thematically linked, we made a higher degree of sorting of the 9 code groups into 5 main themes. By inductively developing the five themes, we were able to maintain empirical detail as well as structuring of the results of the research project [11] into coherent and reportable units. This article is based on one of these main themes: in which situations health personnel find VC useful when approaching patients in psychiatric emergencies. The four situations described in the result section define the scope of usefulness of VC in psyciatric emergencies. The empirical-analytical argument was developed on basis of codes and code groups. Interview excerpts were chosen and included in this article to illustrate the identified themes. By iterative processes both on code level (is the code precisely named to empirical signification?) and code group level (is the code group coherent and covering all it’s codes?), the SDI approach maintains tight connection between empirical data, analysis and interpretation. Hence, validity is maintained through a form of ‘theoretical sampling’ known from Grounded Theory [17].

### Ethics

The study was approved by the Regional Committee for Medical Research Ethics in Norway. Informed consent was obtained from all individual participants included in the study. The data was anonymized, and excerpts from interviews were carefully chosen in order to conceal unnecessary details. For further anonymization, both the nurses and the patients are described in the feminine gender.

## Results

We identified four types of situations in which the health personnel described VC as a useful and important tool in psychiatric emergencies: 1) when there was uncertainty regarding the degree of illness or the level of treatment, 2) when there was a need to clarify the severity of the patient condition, 3) when there was a need to build an alliance with the patient, and 4) when there was disagreement either between health personnel or between the patient and health personnel. These topics are further detailed below.

### Uncertainty

Patients were typically referred to the RPC by a general practitioner or they could come there on their own. After the initial assessment by a nurse, a decision had to be made on further treatment or care. Such decisions involved questions regarding admission or not, and to which level of care the patient should be admitted. At times the RPC nurses made such decisions on their own, but they could also contact a psychiatrist to talk the case over. The nurse suggested VC if she felt uncertain on her own assessments.

“There was substance abuse and suicidal intent. [...] At first I didn’t consider admission, but she was restless and there were children involved. I thought it was important to calm the situation at home, and I needed help [through VC] in order to consider admission.” (Nurse)

During a VC consultation, the patient, the psychiatrist, and the nurse could communicate with each other and the psychiatrist could observe the patient. When the psychiatrists were uncertain on how to handle the patient, they could use VC to gain more information than was possible during telephone calls.

“Especially in those situations where the nurse is very ambivalent or uncertain and I am not able to get a good image of the problem or the patient, I have to enter the case to a much greater degree.” (Psychiatrist)

Telephone calls were used for communication between the nurse and the psychiatrist in most cases, and they explained they did not use VC when they were able to handle the situation confidently.

“When I feel reasonably confident in my own assessment and know the condition is less severe, I have not felt any need to propose VC.” (Nurse).

Thus, telephone calls were preferred when nurses and psychiatrists felt confident in their assessment, while VC was used to bring the psychiatrist into the consultation room at the RPC and thereby reduce uncertainty.

### Severity

A great portion of the VC consultations was used for assessing the degree of severity of the mental illness, especially for patients with suicidal intent or psychotic patients. It seemed like nurses and psychiatrists agreed that potentially severe conditions could add to the uncertainty in the situation, and that it was useful to bring the psychiatrist to the patient in such circumstances.

“A specific reason to use VC is when I want the psychiatrist to assess the degree of severity.” (Nurse)

“Especially when I am uncertain regarding suicidal intent, I find it very important to have a second opinion from the psychiatrist.” (Nurse)

The experience and formal competence of the psychiatrists were often required when nurses believed the condition could be more severe. When using VC, the psychiatrists could both observe and communicate with the patient, and the nurses were not left alone in their own judgment. The nurses suggested that this was important if the outcome of misjudgements was potentially serious, a view that was also shared by psychiatrists.

“At times the nurses are uncertain on the assessment of the patient [...] and if the severity of the condition is such that we should admit the patient, if admission should be at the RPC or at the acute ward in Tromsø.” (Psychiatrist)

Assessing the severity of the condition was important for deciding if and how to admit the patient. Such evaluations are often difficult, and decisions depend on many factors, including the patient condition, cultural background, home environment, and the resources currently available at the RPC.

“At times there has been uncertainty at the RPC about how to assess the patient: for example, whether the patient is psychotic, whether the degree of illness requires hospitalization, or if it would be proper to admit the person at the RPC. This was clarified [when we used VC] for an asylum seeker on the border of being psychotic, where we together got a good understanding of her condition. The way the patient communicated justified admittance to the RPC.” (Psychiatrist)

Therefore, it becomes evident that some assessments are best made in teams, and that VC can be used to support team communication, allow for team building and provide a technical platform for such collaborative assessment.

### Alliances

VC was also found useful for building alliances with patients. This is important as trust between the patient and health providers is necessary in order to improve compliance, and especially in psychiatric emergencies where distrust is part of the disease.

“There was a woman who was manic, and we were very uncertain whether one would be able to attend to her locally or if she had to be involuntarily admitted to Tromsø. We managed through the VC to get an alliance. [...] We agreed that she could be on a voluntary admission at the RPC, and I ordered the medication she needed to calm her down and get her to sleep. Therefore, it is also in such “tipping situations” that one can better handle the patient through voluntary admission. The VC made us confident that it was okay to admit the patient to the RPC ward.” (Psychiatrist)

In this case, the interactions between the psychiatrist, the nurse and the patient was improved through VC and influenced on how the patient was handled. Of course, building an alliance with the mentally ill may not always be possible, but VC may be used as a tool for exploring potential alliances with a critically ill patient:

“The patient was suicidal, exhausted, had not slept, and in crisis. We tried to clarify her condition by using VC. Before the VC consultation, we thought it was possible to build an alliance with her if she was admitted to the RPC. Fortunately, I talked with her through the VC. We were then better able to understand that she was suicidal, and she said that this was her last day. She was thankful for us offering help, but she just wanted to end her life. We offered her medicine, a bed, and a new consultation the next morning. She listened to us, but refused. She did not want admission; it would not help her. She would take her life. [...] She was then involuntarily admitted.” (Psychiatrist)

The failure to build an alliance with this patient was used as important clinical information when deciding how to proceed with the case. These cases illustrate that the combination of VC and a remote psychiatrist works as a replacement for a psychiatrist present in the RPC.

### Disagreement

Finally, the health personnel found VC useful to solve situations when there were conflicting opinions regarding treatment or admission. Patients had often been in contact with more than one health professional prior to the contact with the RPC. At times, these had different opinions in questions of assessment and further care of the patient. In such situations, a VC consultation was useful, as the psychiatrist could make his own assessment and make a final decision without further delay. In one case, the patient was referred by a general practitioner (GP) to the RPC because of suspected suicidal intent, but the nurse did not consider admittance necessary. The nurse asked the psychiatrist to see the patient for further assessment.

“I did not find sufficient reason to admit the patient, but the GP had hinted about suicidal intent. [...] Then we did VC to confirm that we would not admit him and we sent him home. [...] Rather, we did daily follow-ups.” (Nurse)

The nurse at the RPC found it difficult to contradict the opinion of the GP, although she was most likely more used to psychiatric emergencies than the GP. VC was used for the psychiatrist to make his own assessment. During the VC an alternative approach was suggested; the psychiatrist agreed with the nurse’s assessment and recommended daily follow-ups of the patient by the RPC’s ambulant team.

In another case, a patient was discharged from the RPC ward against her will, based on assessment done by a local psychologist. Later that day, the patient contacted a GP who concluded with requesting the RPC for a new assessment and maybe re-admittance. The nurse at the RPC asked for a VC consultation with the psychiatrist to assess the patient’s condition more thoroughly, in order to solve the situation with differing opinions:

“I thought the patient should be re-admitted, but a psychologist had concluded differently. [...] One of our leaders suggested not to overrule the psychologist. I felt we could not allow for this consideration because the patient’s condition was poor. I therefore suggested VC for a new assessment from a psychiatrist.” (Nurse)

The result of the following VC consultation with the psychiatrist was re-admission of the patient to the RPC for several weeks. This case illustrates the complexity of the situations of some patients with mental illness, and that there is a need to enter these situations in a different way than would be possible through only telephone calls.

Situations were also mentioned where VC with the psychiatrist had been used to settle situations where patients and nurses had different perceptions on decisions or treatment options. In one case, a patient admitted to a RPC ward wanted herself discharged after a few days, but the nurses’ assessment indicated further treatment was necessary. VC had been used earlier to the admission, therefore the nurses requested a new VC consultation with the same psychiatrist. As explained by the psychiatrist who assessed the patient through the VC, the consultation ended with the patient changing his mind about admission:

“After a few days of admission, a patient became uneasy and was about to leave and I was asked if I would try to talk to her again. I experienced again that I communicated well with her. We agreed that she should continue to stay for a few days to see if things improved. [...] She was definitely too ill to cope with her condition. If she had been discharged we would have had to inform the police to bring her in for involuntary hospitalization. However, she ended up being voluntarily admitted to the RPC for a long time, and she was discharged normally. It worked well.” (Psychiatrist)

The last text excerpt illustrates the usefulness of VC also when there is a combination of uncertainty, severity, disagreement and need for building an alliance with the patient.

## Discussion and conclusion

In this study, we interviewed psychiatrists and nurses who had used VC consultations in addition to telephone consultations for psychiatric emergencies. The nurses were co-located with patients in RPCs while the psychiatrists were on-call, serving several RPCs from a distance, either from the hospital or from their home. Telephone was used between nurses and psychiatrists, while VC consultations also included the patient. VC consultations with patients were useful for 1) reducing uncertainty among health personnel, 2) assessing the degree of severity of the psychiatric emergencies, 3) building alliances between health personnel and patients, and 4) resolving disagreement regarding treatment and patient admission. In most cases, however, nurses and psychiatrists were confident that they were able to handle the situation sufficiently without the use of VC.

With the introduction of the VC system for psychiatric emergencies, no instructions were given about the situations in which the system should be used. It meant that it was left to the phone-mediated negotiation between local nurses and centrally located psychiatrists to settle whether the use of VC would add to the quality or efficiency of the assessment. The VC system was permanently installed and personnel trained in order to reduce barriers to the use of the system. However, the easiness of telephone calls may nevertheless have kept VC to a minimum. The telephone is the obvious affordance [18, 19] and is taken for granted as synchronous communication at a distance. We have seen in our analysis that VC in psychiatric emergencies has great potential, but provide a limited set of affordances compared to the telephone. Some of these affordances are a priori obvious, for instance for the psychiatrist to observe patients as they speak. Other affordances are not necessarily that obvious, for instance that of patients coming into a position in which they can listen in on conversations between the nurse and the psychiatrist, hence getting to another level of experienced participation. In a previous paper, we reported how patients experienced a stronger confidence that the right decisions were made and improved patient compliance to the psychiatrist orders when VC was used [12]. Some patients were actively commenting and discussing the situation, which led to a greater patient involvement in decision-making for diagnoses and treatment. Patients found this gave them confidence and a better understanding of the importance of following the treatment decided by the psychiatrist. By applying the affordance perspective on VC in acute psychiatric emergencies, we put emphasis on how VC becomes a tool in specific situations, where telephone calls are insufficient. Even if telephone calls seems to be sufficient in most cases it does not imply that VC is superfluous.

In emergencies, decisions need to be made quickly in spite of complexity, uncertainty, lack of information, and distance between patients and health personnel. In psychiatry, early contact directly with a psychiatrist may clarify treatment and admission options and resolve questions about the patient’s degree of severity. A telephone call from the local nurse to a psychiatrist may not sufficiently convey the patient’s situation in such a way that firm decisions can be made. The alternative will often be to admit the patient to the nearest hospital with a psychiatric ward, which will be an extra burden for many patients. When VC is used as an alternative to telephone calls, the patient can be seen and heard and all participants can be included in the decision. For medical emergencies, VC has been shown to improve the quality of care and the confidence of professionals and lay persons in complex or challenging situations [20, 21]. In one study, doctors reported becoming more involved in the treatment at the remote hospital when they could see the patient and they indicated that VC helped establish a shared interpretation of the situation [22]. These studies support our findings that VC can be a useful tool for more involved decision-making in complex or challenging situations when there are several participants and time is limited.

VC has been shown to build the same level of trust in groups as face-to-face communication, while audio-only communication is inferior for building trust among team members [23]. Trust is important for cooperation, both for patients and teams of health personnel. When VC-consultations were used for psychiatric emergencies, patients became more involved in decision-making because of direct contact with the psychiatrist, while health personnel indicated improved confidence, reduced uncertainty, and an increase in shared responsibilities for decisions [12]. Thus, VC can be superior to telephone calls when there is a need for team processes in order to create trust among participants and confidence in the decisions that are made. Our participants indicated that the back-and-forth interactions during VC consultations would not have been possible over the phone. However, even though VC enables richer communication between participants, it does not have to be used all the time, since traditional telephone calls are useful for straightforward cases.

VC was used as a tool to establish a decentralized 24/7 health service for psychiatric emergencies, and affords an essential organizational change: For many patients a VC consultation initiated treatment at the RPC instead of needing an admission to the acute psychiatric hospital ward several hours away. If the condition of locally admitted patients deteriorated, a psychiatrist would be easily available for re-assessment of the patient. The VC-mediated availability of the psychiatrists helped to ensure quality in these rural and sparsely populated areas. This is in accordance with previous studies indicating that VC in medical emergencies has reduced the need for patient transport [24–26], improved the quality of clinical treatment [27–29], and helped reduce geographical differences in trauma treatment [30].

This study was conducted one to two years after the VC system became operational. As is common with most new systems and organizational changes, adoption is slow in the beginning. Some of the nurses had therefore participated in only one VC session, while psychiatrists had used VC several times. Both psychiatrists and nurses were having positive expectations about the use of VC with patients in psychiatric emergencies, although our study was conducted at an early stage of VC adoption. In interviews, participants indicated they could probably have tried to use VC with more patients, but they had only used VC in situations where they were quite certain it would prove useful. Further studies are needed to explore if there are psychiatric emergencies in which VC should not be used.

The richness in communication provided by VC improves the ability to develop and maintain collaboration in teams across distance. In psychiatric emergencies, a psychiatrist that would otherwise be unavailable for a direct patient contact can be brought into a consultation at remote sites through VC. This study shows that VC is useful in psychiatric emergencies for reducing uncertainty, assessing the degree of severity of patient illness, building alliances between health personnel and patients, and for resolving disagreement regarding treatment and patient admission. VC not only affords another level of participation and patient involvement, but may with a longer period of use make ground for better organizational models and more effective logistics when time is limited. While this study has been looking into the application of VC in acute psychiatry at a very early stage, our prudent conclusion is that VC would be useful for many challenging psychiatric emergencies and suitable for increasing quality in decentralized psychiatric services. Studies after a longer period of adoption should be carried out to understand the broader potential of VC for health-related emergencies.

## Abbreviations

RPC: Regional Psychiatric Center
VC: Videoconferencing
GP: General Practitioner
